# Supplementary material for: Timing of TORC1 inhibition dictates Pol III involvement in Caenorhabditis elegans longevity
Source: Life Sci Alliance. 2024 May 13;7(7):e202402735. doi: 10.26508/lsa.202402735 (PMC11091362; doi:10.26508/lsa.202402735)
Supplement: Supplementary file 1 [file LSA-2024-02735_TableS1.docx]

**Table S1**

| **Figure** | **Trial** | **Strain** | **Genotype** | **Mean Lifespan (days)** | **Extension (%)** | ***p* value (Log-rank) vs** | | ***N* dead**  **(total)** |
| --- | --- | --- | --- | --- | --- | --- | --- | --- |
| S3 A | 1 | WT | Control RNAi | 12.68 |  |  | 79 | |
|  |  | WT | *rpc-1* RNAi | 12.91 | 1.8 | WT cont.<0.0001 | 87 | |
|  |  | *rsks-1(ok1255)* | Control RNAi | 15.68 |  |  | 88 | |
|  |  | *rsks-1(ok1255)* | *rpc-1* RNAi | 16.86 | 33.0 | *rsks-1(ok1255)* cont.<0.05 | 80 | |
|  | 2 | WT | Control RNAi | 10.77 |  |  | 76 | |
|  |  | WT | *rpc-1* RNAi | 11.89 | 9.42 | WT cont<0.0001 | 85 | |
|  |  | *rsks-1(ok1255)* | Control RNAi | 16.08 |  |  | 69 | |
|  |  | *rsks-1(ok1255)* | *rpc-1* RNAi | 17.21 | 6.57 | *rsks-1(ok1255)* cont<0.05 | 76 | |
| S3 B | 1 | WT | Control RNAi | 12.32 |  |  | 103 | |
|  |  | WT | *rpc-1* RNAi | 13.42 | 17.2 | WT cont.<0.0001 | 100 | |
|  |  | *ppp-1* | Control RNAi | 13.65 |  |  | 98 | |
|  |  | *ppp-1* | *rpc-1* RNAi | 14.72 | 8.06 | *ppp-1 cont.*<0.05 | 94 | |
|  | 2 | WT | Control RNAi | 11.97 |  |  | 89 | |
|  |  | WT | *rpc-1* RNAi | 12.99 | 11.10 | WT cont.<0.0001 | 92 | |
|  |  | *ppp-1* | Control RNAi | 13.10 |  |  | 54 | |
|  |  | *ppp-1* | *rpc-1* RNAi | 14.11 | 11.03 | *ppp-1 cont.*<0.05 | 51 | |
| S3 C | 1 | WT | Control RNAi | 11.23 |  |  | 54 | |
|  |  | WT | *rpc-1* RNAi | 13.16 | 17.2 | WT cont<0.0001 | 62 | |
|  |  | *ife-2(ok306)* | Control RNAi | 11.52 |  |  | 46 | |
|  |  | *ife-2(ok306)* | *rpc-1* RNAi | 13.74 | 22.0 | *ife-2(ok306)* cont.<0.0001 | 31 | |
|  | 2 | WT | Control RNAi | 10.69 |  |  | 89 | |
|  |  | WT | *rpc-1* RNAi | 11.83 | 9.64 | WT cont<0.003 | 85 | |
|  |  | *ife-2(ok306)* | Control RNAi | 12.45 |  |  | 81 | |
|  |  | *ife-2(ok306)* | *rpc-1* RNAi | 14.03 | 11.26 | *ife-2(ok306)* cont<0.0001 | 78 | |
