# Supplementary material for: Timing of TORC1 inhibition dictates Pol III involvement in Caenorhabditis elegans longevity
Source: Life Sci Alliance. 2024 May 13;7(7):e202402735. doi: 10.26508/lsa.202402735 (PMC11091362; doi:10.26508/lsa.202402735)
Supplement: Supplementary file 2 [file LSA-2024-02735_TableS2.docx]

**Table S2**

| **Figure** | **Trial** | **Strain** | **Genotype** | **Mean Lifespan (days)** | **Extension (%)** | ***p* value (Log-rank) vs** | ***N* dead**  **(total)** |
| --- | --- | --- | --- | --- | --- | --- | --- |
| S4 A | **1** | WT | Control RNAi | 11.49 |  |  | 87 |
|  |  | WT | *rpc-1* RNAi | 14.0 | 21.8 | WT cont.<0.0001 | 90 |
|  |  | *daf-2(m577)* | Control RNAi | 23.49 |  |  | 71 |
|  |  | *daf-2(m577)* | *rpc-1* RNAi | 26.21 | 128.1 | *daf-2(m577)* cont.<0.05 | 74 |
|  | **2** | WT | Control RNAi | 11.11 |  |  | 71 |
|  |  | WT | *rpc-1* RNAi | 13.01 | 14.60 | WT cont<0.0001 | 98 |
|  |  | *daf-2(m577)* | Control RNAi | 22.45 |  |  | 81 |
|  |  | *daf-2(m577)* | *rpc-1* RNAi | 25.97 | 13.55 | *daf-2(m577)* cont<0.05 | 75 |
| S4 B | **1** | WT | Control RNAi | 12.14 |  |  | 59 |
|  |  | WT | *rpc-1* RNAi | 13.19 | 8.6 | WT cont.<0.05 | 44 |
|  |  | *glp-1(e2141)* | Control RNAi | 13.27 |  |  | 94 |
|  |  | *glp-1(e2141)* | *rpc-1* RNAi | 14.33 | 18.0 | *glp-1(e2141*) cont. <0.05 | 56 |
|  | **2** | WT | Control RNAi | 10.54 |  |  | 93 |
|  |  | WT | *rpc-1* RNAi | 11.56 | 8.82 | WT cont.<0.0001 | 98 |
|  |  | *glp-1(e2141)* | Control RNAi | 13.68 |  |  | 77 |
|  |  | *glp-1(e2141)* | *rpc-1* RNAi | 14.95 | 8.49 | *glp-4(bn2)* cont<0.05 | 83 |
| S4 C | 1 | WT | Control RNAi | 12.31 |  |  | 113 |
|  |  | WT | *rpc-1* RNAi | 13.33 | 17.2 | WT cont.<0.0001 | 105 |
|  |  | *glp-4(bn2)* | Control RNAi | 13.66 |  |  | 165 |
|  |  | *glp-4(bn2)* | *rpc-1* RNAi | 14.72 | 15.3 | *glp-4(bn2)* cont. <0.05 | 130 |
|  | 2 | WT | Control RNAi | 11.30 |  |  | 79 |
|  |  | WT | *rpc-1* RNAi | 12.53 | 9.82 | WT cont.<0.0001 | 91 |
|  |  | *glp-4(bn2)* | Control RNAi | 13.66 |  |  | 88 |
|  |  | *glp-4(bn2)* | *rpc-1* RNAi | 14.98 | 8.81 | *glp-4(bn2)* cont<0.05 | 82 |
